# Phage Therapy as an Alternative Strategy Against *Pseudomonas aeruginosa*: A Narrative Review of Preclinical and Clinical Evidence

**DOI:** 10.1002/mbo3.70303

**Published:** 2026-05-03

**Authors:** Gustavo Aparecido da Cunha, Raine Piva Amaral, Ana Luisa Cauvila dos Santos, Bárbara Rafaela Ferreira Aio, Maria Fernanda Romboli Durante, Luiz Cosme Cotta Malaquias, Gabriel Magno de Freitas Almeida, Luiz Felipe Leomil Coelho

**Affiliations:** ^1^ Department of Microbiology and Immunology Federal University of Alfenas Alfenas Minas Gerais Brazil; ^2^ The Norwegian College of Fishery Science, Faculty of Biosciences, Fisheries and Economics UiT ‐ The Arctic University of Norway Tromsø Norway

**Keywords:** antimicrobial resistance, bacteriophage, multi‐drug‐resistance, phage therapy, *Pseudomonas aeruginosa*

## Abstract

*Pseudomonas aeruginosa* is an opportunistic pathogen and one of the main causes of nosocomial infections. This pathogen affects immunocompromised individuals such as patients with chronic wounds or cystic fibrosis. Its capacity to evade antimicrobial therapies through resistance mechanisms has resulted in widespread prevalence of multidrug‐resistant (MDR), extensively drug‐resistant (XDR), and pandrug‐resistant (PDR) strains. We searched three databases (PubMed/Medline, Scopus, and Web of Science) for articles evaluating the effects of phage administration in the management of *P. aeruginosa* infections in animals and humans. We analyzed the main characteristics of the interventions, the coverage of different bacterial strains, and discussed possible gaps in the evidence available in the last 10 years. Literature shows strong evidence that the use of phage therapy for several clinical conditions in humans and animal models is a safe and effective intervention for infections caused by MDR/XDR/PDR *P. aeruginosa*. Despite its therapeutic potential, phage therapy still faces several limitations such as lack of standardized dosing protocols, inconsistent endotoxin quantification, and limited regulatory frameworks. Future guidelines should focus on the regulation and validation of phage therapy in clinical practice, after overcoming the limitations currently identified. Further studies should ensure the standardization of phage production and the validation of delivery systems and routes of administration. Conducting multicenter clinical trials can contribute to the clinical implementation of phage therapy in countries where it is not yet regulated.

## Graphical abstract

Phage therapy shows promising efficacy against MDR, XDR, and PDR *Pseudomonas aeruginosa* infections across different models and administration routes. Evidence highlights reduced mortality, bacterial clearance, and synergy with antibiotics, while emphasizing the need for standardized protocols and advanced delivery systems to support clinical translation.

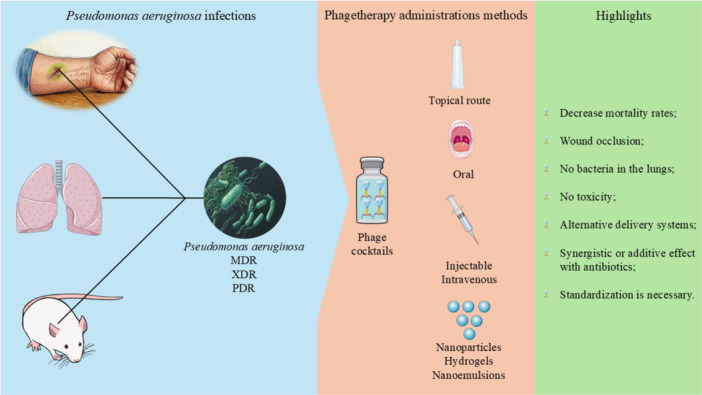

## 1 Introduction

### 1.1 Pseudomonas aeruginosa

*Pseudomonas aeruginosa* is a Gram‐negative bacillus commonly found in hospital environments. It is a highly adaptable microorganism that colonizes biotic and abiotic surfaces, and it is intrinsically resistant to a wide range of antibiotics (Chegini et al. 2020; Del Giacomo et al. 2022). This bacterial pathogen is responsible for 7% of all nosocomial infections worldwide and is a leading cause of mortality from respiratory tract and bloodstream infections (Magill et al. 2014; Ng et al. 2023; Sathe et al. 2023; Shi et al. 2019; Weiner et al. 2016). In general, mortality rates among hospitalized individuals reach 34% in infections with antibiotic‐resistant *P. aeruginosa* strains (Nathwani et al. 2014).

Several virulence factors presented by *P. aeruginosa* contribute to the course of the infectious process and to the evasion of the host's immune response and resistance to antibiotics. For example, the OprF porin present in the outer membrane of *P. aeruginosa* controls the adhesion process to the human alveolar epithelium, the osmotic gradient, ionic permeability, and ensures phagocytic evasion by modulating the expression of type III secretion system (T3SS) genes (Jurado‐Martín et al. 2021; Moussouni et al. 2021; Yaeger et al. 2024). The various isoforms of the siderophore pyoverdine produced by *P. aeruginosa* act in the process of iron absorption, biofilm formation, and in the catalysis of toxins such as Exotoxin A, which inhibits protein synthesis and induces cell death (Jurado‐Martín et al. 2021; Schalk et al. 2001; Schalk and Guillon 2013). Due to this virulence factors, *P. aeruginosa* cells can adapt to distinct microenvironments as wounds, lungs and bones (Elfadadny et al. 2024; Gupta et al. 2019; Hahn et al. 2023; Y. Lin et al. 2019; Patel et al. 2021).

The resistance mechanisms described in *P. aeruginosa* are distributed among the various strains of this bacillus, resulting in organisms with distinct genetic patterns and, in many cases, making them insensitive to all classes of available antibiotics. The classification proposed by Magiorakos et al. (2012) classifies bacterial pathogens found in public health as multidrug‐resistant (MDR), extreme drug‐resistant (XDR), and pan‐drug‐resistant (PDR). Clinical isolates of *P. aeruginosa* are classified as multidrug‐resistant (MDR) when they exhibit non‐susceptibility to at least one agent in three or more antimicrobial classes. Extensively drug‐resistant (XDR) isolates are defined as non‐susceptible to at least one agent in all but two or fewer antimicrobial classes, remaining susceptible to only one or two classes. Finally, isolates that are non‐susceptible to all agents in all antimicrobial classes tested are classified as pandrug‐resistant (PDR). (Cosentino et al. 2023; Magiorakos et al. 2012).

The European Union reports that 30% of clinical isolates of *P. aeruginosa* described in 2020 were MDR and that 18% did not respond to last‐line antibiotics such as carbapenems (World Health Organization and the European Centre for Disease Prevention Control 2022). Data obtained by Abdelaziz et al. (2024), from isolates from hospitals in Egypt describe that 76% of the strains analyzed were XDR and that genes producing carbapenemases, extended‐spectrum β‐lactamases [ESBLs], and Metallo β‐lactamase (MBL) were observed in 73.6%, 75.5%, 88.7% of the XDR strains of *P. aeruginosa* analyzed, respectively (Abdelaziz et al. 2024). Due to the significant increase in the number of infections caused by *P. aeruginosa*, the high mortality rates and the antibiotic resistance patterns, new therapeutic strategies are required to control infections caused by this pathogen (Aghaee et al. 2021; S. Liu et al. 2022; Montero et al. 2020; World Health Organization 2024).

### 1.2 Phage Therapy

Among the potential approaches to control *P. aeruginosa* infections, phage therapy stands out for presenting significant antimicrobial effects and mechanisms of specificity to a single bacterial host. Phage therapy involves the application of bacterial viruses to treat bacterial infections; these viruses are called bacteriophages or phages (derived from the Greek meaning “bacteria‐eater”) (Strathdee et al. 2023; Summers 2012). In summary, phages can recognize receptors on the bacterial membrane and bind to them. Next, pores form in the cell wall (disruption of peptidoglycan) and viral genetic material is inserted. The viral genome is integrated into the bacterial genome and new virions are produced. After maturation, the host bacterium lyses and the cycle restarts. This technique was first described in the early 20th century by the microbiologist Felix d'Herelle, who described the bacteriolytic activity performed by this type of virus and used bacteriophage preparations to treat children suffering from bacterial dysentery (Summers 2012).

Due to the success of the therapy, phage cocktails were widely used to treat bacterial infections in humans and animals until the 1940s. However, after the introduction of penicillin to the market in 1940, phage therapy lost popularity (Almeida and Sundberg 2020) in the Western Countries (Kaur et al. 2021; Summers 2012) while it remained a reality inside the Soviet Union and Poland. It is important to note that phage therapy brings together benefits that increase its versatility compared to antibiotics and it has renewed interest in this intervention within the Western scientific community since the last decades (Altamirano and Barr 2019). Genetic diversity, abundance, and ubiquity make phage an extensive source of antimicrobial particles (Summers 2012), including those targeting *P. aeruginosa*.

Although out of the scope of this review, it is important to note that phage therapy is currently being employed against *P. aeruginosa* in compassionate cases worldwide (McCallin et al. 2019) and already is a reality in clinics inside Belgium (Pirnay et al. 2024). Of the first hundred consecutive cases in Belgium, 49 involved *P. aeruginosa* infections, of which 37 resulted in clinical improvement. Therefore, this article is presented as a narrative review aiming to integrate preclinical and clinical evidence on the use of bacteriophages against *Pseudomonas aeruginosa* infections. Rather than performing a systematic comparison of interventions, we sought to contextualize therapeutic strategies, experimental models, delivery routes, and outcomes reported across heterogeneous studies, highlighting trends, gaps, and translational challenges. The literature was surveyed using PubMed, Scopus, and Web of Science, focusing on studies published between 2010 and 2025. Preclinical studies, clinical trials, and well‐documented case reports addressing bacteriophage therapy against *P. aeruginosa* were considered. Given the narrative nature of this review, no formal exclusion criteria or quantitative synthesis were applied, and studies were selected based on relevance to infection models, therapeutic strategies, and translational insights.

## 2 Use of Phage Therapy to Treat *P. aeruginosa Infections*

Here, we summarized the state‐of‐the‐art of phage therapy in some clinical conditions (infected wounds, lung infections) in animal models and in reported clinical applications. All studies addressing all types of infections described in this review reveal a wide diversity of experimental designs, reflecting the absence of standardized protocols and the context‐dependent nature of phage therapy applications. Additionally, we found case reports of the effects of phage therapy on osteomyelitis caused by *P. aeruginosa*. and retrieved additional studies with alternative animal models, such as *Galleria mellonella* and *Danio rerio*, that evaluated the outcomes of systemic infection in these animal models.

Phage therapy against MDR *P. aeruginosa* has shown promising results in a wide range of infectious conditions caused by this bacillus in preclinical studies, case reports and clinical trials (Can et al. 2018; Fukuda et al. 2012; Jault et al. 2019; Teney et al. 2024). The application of viral cocktails by inhalation promotes a reduction in the bacterial load in patients with cystic fibrosis and pneumonia (Hahn et al. 2023; Köhler et al. 2023). Some authors suggest that the association of phages with reference antibiotics promotes better results and reduces the possibility of bacterial resistance due to the multiple mechanisms of aggression generated by the combination of both therapies (Duplessis et al. 2021; Köhler et al. 2023). The association of inhaled/injectable phages with antibiotics (imipenem‐relebactam) promoted the improvement of the infected graft and the respiratory condition of a patient carrying XDR *P. aeruginosa* (Teney et al. 2024). Additionally, the beneficial effects associated with the combination of phages with antibiotics are not related to serious immunological complications. Few studies suggest any toxic effect of the cocktails or of their combination with antimicrobial drugs (D. Liu et al. 2021). Some reports of toxicity appear to be associated not with viral components, but rather with the presence of bacterial epitopes such as endotoxins (Yang et al. 2021).

**Figure 1:**
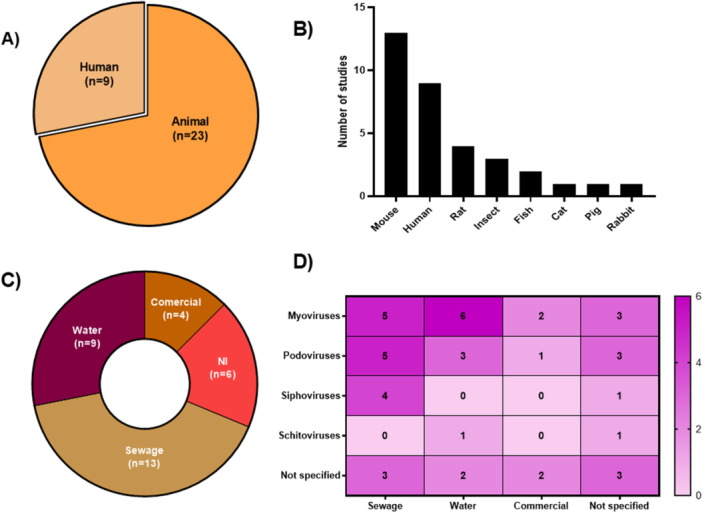
Overview of the phage therapy studies against *Pseudomonas aeruginosa*. (A) Proportion of studies conducted in animal models (blue) versus human subjects (red). (B) Number of host organisms employed across the included studies. (C) Distribution of the sources used for phage isolation. (D) Heatmap showing the correlation between phage family's groups and their respective sources of isolation.

In general, most studies use phages that were used alone, in a cocktail with multiple phages, or associated with antibiotics (Table 1). Across the 32 eligible publications, 23 (72%) were conducted in experimental animal models, whereas 9 (28%) involved human subjects (Figure 1A). Among the eukaryotic hosts used in phage therapy (Figure 1B), mice were the most frequently used organism (*n* = 13), followed by humans (*n* = 10) and rats (*n* = 4). Alternative and low‐cost models such as *Galleria mellonella* larvae (*n* = 3) and zebrafish (*n* = 2) were also used, while large mammals (cat, pig, rabbit) appeared only once each.

**Table 1:** Selected preclinical and clinical studies reporting the use of bacteriophage therapy against *Pseudomonas aeruginosa* infections.

Ref.	Dose*	Route	Disease	Administration	Source of isolation	Outcomes
(Ménard et al. (2021))	10^9^ PFU/mL	Topic	Diabetic wound	Cocktail	Sewage water	− Wound healed − Decrease inflammatory markers (TNF‐α, NF‐κβ, IL‐1β, IL‐8, and MCP‐1)
(Olorundare et al. (2024))	[‐]	Oral	Diabetic wounds and surgical wounds	Cocktail	Purchase	− Wound healed − Atoxicity − No patient deaths
(Cosentino et al. (2023))	10^9^ PFU/mL	Topic	Chronic wounds	Cocktail	Water	− Wound healed − Sterility of open wounds − Atoxicity
(Pires et al. (2020))	10^8^ PFU/mL	Gauze topic	Skin wound	Single and cocktail	Sewage water	− No animals deaths − Better healing than ceftriaxone
(Patel et al. (2021))	10^8^ PFU/mL	Topic	Skin thermal injury	Single and cocktail	Sewage water	− Decrease mortality − Wound healed − reduction in inflammatory cell infiltrate
(McCallin et al. (2019))	10^10^ PFU/mL	Topic	Skin wound	Cocktail	Sewage water	− Improve wound‐healing process − Decrease bacterial load
(Olszak et al. (2015))	10^9^ PFU/mL	Topic	Diabetic wound	Cocktail	Sewage water	− Decrease bacterial colony counts − Improve wound‐healing − Combination with gentamicin enhances healing
(Pirnay et al. (2024))	10^9^ PFU/mL	Topic	Skin wound	Single	Sewage water	− Complete elimination of bacterial infection − Wound healed
(S. Mabrouk et al. (2022))	10^9^ PFU/mL	Topic	Surgery wound	Single	Water	− Wound healed (previously open for five months) − Decrease of bacterial load
(Mendes et al. (2013))	10^7^ PFU/mL	Topic	Burn wound	[‐]	Sewage water	− Improve wound‐healing
(Duplessis et al. (2021))	10^9^ PFU/mL	Gauze topic	Skin wound	Single and cocktail	Water samples	− Wound healed
(Piranaghl et al. (2023))	10^7^ PFU/mL	Topic	Skin wound	Single and cocktail	Water samples	− Reduced significantly the bacterial load − Rapid wound healing without any side effects − Synergistic effect with ciprofloxacin accelerated − healing
(Egido et al. (2022))	10^10^ PFU/mL	Inhalation	Cystic Fibrosis	Single	[‐]	− Decrease bacterial load
(Kavanagh and Sheehan (2018))	10^9^ PFU/mL	Inhalation	Chronic lung infection	Single	[‐]	− Patient's clinical − condition improved rapidly − On day 5 post‐treatment initiation, the − patient was free from respiratory obstruction − ‐Stable bilateral lower lung condensations
(Liu et al. (2022))	10^9^ PFU/mL	Inhalation	Pneumonia	Single and cocktail	Sewage water	− Reduction of bacterial load − Improve survival rate
(Köhler et al. (2023))	10^9^ PFU/mL	Intraperitoneal and Intratracheal	Pneumonia	Single and cocktail	Wastwater	− Improve survival rate − Reduction of bacterial load
(Lin et al. (2019))	10^11^ PFU/mL	Inhalation	Pneumonia	Single	Water	− Improve survival rate − ‐Reduction of bacterial load − ‐Reduction of inflammatory cell infiltrate − ‐Reduction of TNF‐α, IL‐1β, and IFN‐γ
(Pliska (2020))	10^9^ PFU/mL	Intratracheal	Pneumonia	Single	Sewage water	− Reduction of bacterial load − Reduction of inflammatory cell infiltrate
(Karthika et al. (2023))	10^8^ PFU/mL	Intraperitoneal	Pneumonia	Single	Purchase	− Reduction of bacterial load − Reduction of TNF‐α, and IL‐6
(Racenis et al. (2022))	10^7^ PFU/mL	Tail vein and Intratracheal	Pneumonia	Single	Hospital water, Farm	− Reduction of bacterial load − Reduction of TNF‐α, and IL‐6 − Reduction of tissue damage
(Liu et al. (2021))	10^4,6,8^ PFU/mL	Intratracheal	Pneumonia	Single	Sewage water	− Reduction of bacterial load − Reduction of TNF‐α, IL‐6 and IL‐1 β
(Saraceni et al. (2016))	10^7^ PFU/mL	Inhalation	Chronic lung infection	Single	[‐]	− ‐Reduction of bacterial load
(Sathe et al. (2023))	10^10^ PFU/mL	Injection	Systemic infection	Single and cocktail	Waters samples	− The phage application showed a significant impact on Galleria larval rescue from lethal infection
(Magill et al. (2014))	10^6^ PFU/mL	Intravenous	Osteomyelitis	Cocktail	[‐]	− No toxic effects − Patient was healed with local and intravenous injections of purified phages
(Schalk and Guillon (2013))	10^8^ PFU/mL	Injection	Systemic infection	Cocktail	Sewage water	− Decrease lethality, ‐Reduction of bacterial burden, − Reduction of inflammatory response
(Magiorakos et al. (2012))	10^9^ PFU/mL	Dispersion in containers	Systemic infection	Cocktail	Sewage water	− No toxicity − Improve survival rate − Decrease of tissue damage
(Schalk et al. (2001))	5×10^1,2,3,4^ PFU/mL	Injection	Systemic infection	Single	Sewage water	− Reduction of bacterial load − No improvement in survival rate
(Pereira et al. (2020))	10^8^PFU/mL	Direct and compress	Osteomyelitis	Cocktail	Purchase	− Reduction of bacterial load − Improve of tissue reconstruction
(Serrano et al. (2023))	10^11^ PFU/mL	Intravenous	Osteomyelitis	Single	[‐]	− Improve tissue reconstruction − Decrease of C‐reactive protein and White blood cells
(Ng et al. (2023))	10^7^ PFU/mL	Injection	Systemic infection	Cocktail	[‐]	− No adverse effects − Improve survival rate
(Shafigh Kheljan et al. (2023))	10^7^ PFU/mL	Irrigation	Osteomyelitis	Cocktail	Purchase	− Reduction of inflammation − Wound healed − Patient was healed
(Kaszab et al. (2023))	10^8^ PFU/mL	Eye dropping	Keratitis	Single	Water sample	− Reduction of bacterial load − Decrease of MPO levels − Reduction of inflammatory infiltrate − No tissue damage

* Some values adjusted from MOI

Despite the therapeutic approach used (single or cocktail administration) there were significant reductions in bacterial population density and, in some cases, total sterilization of the treated tissue. Administration protocols varied significantly across studies, ranging from single‐dose applications to twice‐daily treatments extending over several weeks. For example, Duplessis et al. (2021) describes that a single administration of phage directly to the lungs via a single intubation‐mediated intratracheal instillation (IMIT) protected mice from lethal infection, whereas Can et al. (2018) applied phage intranasally two and 12 h after the inoculation. These studies suggest the heterogeneity in treatment strategies and highlight the need for standardized dosing protocols in future studies.

Phages were obtained from sewage, wastewater, water samples, or purchased from commercial sources (Figure 1C). Sewage represented the most common source, accounting for 41% of all isolates (*n* = 13), followed by water samples, 28% (*n* = 9). Only 12% of the studies (*n* = 4) used commercially available phage preparations and in 19% (*n* = 6) of the cases the origin of the phages was not specified by the authors. Myoviruses displayed the broadest distribution, with a high number of hits in water samples (*n* = 6) but remaining well represented in sewage (*n* = 5) and commercial collections (*n* = 2). Podoviruses were most abundant in sewage (*n* = 5) and notably less frequent in other sources. Siphoviruses were almost exclusively retrieved from sewage (*n* = 4), whereas schitoviruses were rare and confined to single sources. These studies suggest that environmental origin strongly influences the spectrum of recoverable anti‐*P. aeruginos*a phages, with sewage providing the greatest diversity from a morphological point of view (Figure 1D).

**Figure 2:**
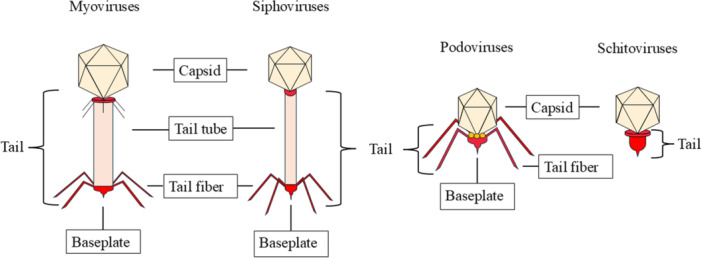
Schematic representation of the main bacteriophages used to treat infections caused by *Pseudomonas aeruginosa*.

Phages were usually administered at concentrations ranging from 10^4^ to 10^11^ PFU/mL. Among the studies that sought to characterize viral particles, we observed the prevalence of viruses with myoviruses, siphoviruses, podoviruses and schitoviruses morphologies, respectively. Figure 2 illustrates the bacteriophage morphotypes reported in studies addressing *P. aeruginosa* infections, without implying preferential efficacy or standardized selection criteria.

There aren't any positive correlation or favorable outcomes for any specific viral group, although the host range appears to be broader myoviruses with an effect on MDR, XDR, PDR and wild strains of *P. aeruginosa* (Can et al. 2018; R. Y. K. Chang et al. 2022; Jeon and Yong 2019; Köhler et al. 2023). In addition to the most frequently reported phage groups, other studies have also isolated and characterized phages from less commonly described groups such as schitoviruses and straboviruses (Braunstein et al. 2024; Ferry et al. 2022; Karthika et al. 2023), indicating a broader virological diversity with therapeutic potential against *P. aeruginosa* infections.

### 2.1 Phage Therapy in the Healing Process of Infected Wounds

Dermal lesions are one of the main sites of colonization for bacterial pathogens due to their difficult healing and the availability of nutrients. *P. aeruginosa* is frequently associated with wound infections and presents a set of unique characteristics such as the greening of the lesion, a pungent sweet odor resulting from the production of pyocyanin, and the emission of fluorescent light under ultraviolet light by the siderophore pyoverdine (Ghssein and Ezzeddine 2022; Schalk and Guillon 2013). This bacterium is a common pathogen in biofilms colonizing wounds of burn, diabetic, and immunocompromised patients (Chegini et al. 2020; Ghanaim et al. 2023; Mendes et al. 2013; Piranaghl et al. 2023). *P. aeruginosa* biofilms play a key role in the progression of chronic wounds due to the production of rhamnolipids, a class of biosurfactants that reduce neutrophil migration and induce tissue necrosis (Berlanga and Guerrero 2016; C. Chang et al. 2022; S. Liu et al. 2022).

There was great diversity among the animal models chosen for wound induction. Rats and mice have consolidated themselves as the main mimetic models in the wound healing process found (Gupta et al. 2019; Mendes et al. 2013; Patel et al. 2021). Some methods have been explored to induce wound formation in preclinical models, including skin removal by scalpel, burning with a hot bar, and diabetic lesions (induction of diabetes in susceptible animals following by scalp removal). Although pigs are considered the models of choice for wound healing trials due to their histological and immunological similarities with humans, only one study evaluated the outcomes of phage therapy on infected wounds in these animals (Mendes et al. 2013). In general, the use of pigs is hampered by high costs, handling and the need for large areas to keep the animals (Sullivan et al. 2001).

Although the administration of phages contributed to the wound‐healing process in treated animals, the effects appear to be directly linked to the elimination of pathogen and not to any biological event resulting from the interaction of the viruses with the treated animals. Therefore, the reduction of lesion size and wound healing process could be directly related to elimination of *P. aeruginosa* infection foci. Although the studies did not observe or quantify direct effects on re‐epithelialization and tissue occlusion, phage cocktails treatment do not affect such outcomes negatively and are considered safe and non‐toxic compounds (Chung et al. 2023; D. Liu et al. 2021). However, it is imperative to make a safe formulation where bacterial endotoxins (Chung et al. 2023) were removed, or its concentration is below the accepted threshold by regulatory health agencies. Few studies have quantified the traces of bacterial endotoxins in the cocktails (C. Chang et al. 2022; Forti et al. 2018).

The quantification of compounds capable of inducing immune responses is crucial, because 25% of wound healing studies have been conducted in humans. In clinical reports, phage therapy has been explored in diabetic patients with wounds, elderly patients and individuals infected with surgical lesions. In these studies, phage therapy has proven to be effective in promoting sterilization and closure of lesions (Gupta et al. 2019; Jokar et al. 2024; Nadareishvili et al. 2020). The usual doses of phage cocktails ranged from 10^5^ to 10^9^ PFU/mL (intervention times of 5 to 115 days). Topical administration was the route of choice for 91% of the studies, and only 9% used for the oral route (Table 1).

In none of the studies were their reports of toxicity, and in many cases, the complete occlusion of the wounds occurred at the end of the intervention time. Nadareishvili et al. (2020), reported that patients with persistent infections and open lesions were cured by oral phage therapy, which showed infectious activity and systemic administration even when ingested. The authors utilized alkaline water as a neutralizing agent for stomach pH due to the sensitivity of bacteriophages to factors such as pH and temperature. This concern is reflected in other studies, where different delivery systems, such as hydrogels, nanoemulsions, and sterile gauzes, were proposed to enhance lytic activity and reduce environmental pressure on viruses (Ferry et al. 2018; S. Mabrouk et al. 2022; Piranaghl et al. 2023; Shafigh Kheljan et al. 2023).

### 2.2 Phage Therapy in Pulmonary Infections

*Pseudomonas aeruginosa* is responsible for pulmonary infections, affecting mainly immunocompromised individuals and those with cystic fibrosis (Hahn et al. 2023; Zhang et al. 2021). Here, preclinical models were preferred to investigate the outcomes of *P. aeruginosa* pulmonary infection in animal models, especially mice. Bacterial infection was done by intranasal or intratracheal routes with 10^6^ CFU/mL to 10^8^ CFU/mL to infect animals.

Mice were the most widely used animal models for the outcomes of lung infection by *P. aeruginosa*. According to Fröhlich (2024), the choice of experimental model for lung infections should consider the immunological and histological patterns of the progression of the disease under analysis. Furthermore, high compatibility with humans is required so that the outcomes can mimic clinical reality. Rodents constitute a satisfactory model for mimicking lung infection by *P. aeruginosa*, but they have limitations such as incompatibility with some pathogens and early death (Fröhlich 2024). BALB/c, C57BL/6 and ICR strains were the most widely used animal models to evaluate the effects of phage therapy on the outcomes of *P. aeruginosa* pulmonary infection (75%). The remaining 25% of studies reported the results of this intervention in humans. Inhalation was the preferred route of administration for 50% of the studies analyzed, followed by intratracheal (40%), and 10% used intraperitoneal inoculation. In all studies, no toxicity or adverse effects were reported after phage administration. Treatment with phages to lung infections varies between 1 and 12 days, with a predominance of 7‐day treatment (40%). Also, it was verified a wide variation in the phage concentrations used in the treatment **bone**, whose PFU/mL were between 10^4^ and 10^10^. It is important to highlight that phage therapy in some studies promoted the sterilization of *P. aeruginosa* in the lungs of treated animals and in case reports.

Unlike the tests with infected wounds, in the lung infection models, the administration of the phage cocktails did not occur with any delivery systems. The main approach was based on the dilution of the viral particles in a buffered saline solution. The intranasal route by direct inhalation or nebulization were preferred in the studies, but in some cases, some studies chose the intraperitoneal and intratracheal routes. In all cases, the administration of phage cocktails was able to reduce the number of multi‐ and pan‐resistant *P. aeruginosa* from the lungs of the animal models (R. Y. K. Chang et al. 2022; Waters et al. 2017; Zhang et al. 2021). Histological findings demonstrate the control of the inflammatory process and the prominent reduction of pro‐inflammatory cytokines IL‐6, TNF‐α, and IL‐1β (R. Y. K. Chang et al. 2022; Chen et al. 2021).

Some case reports have shown promising results against MDR *P. aeruginosa* strains responsible for lung infections in life‐threatening patients (Hahn et al. 2023). Köhler et al. (2023), describes how aerosolized phage therapy promoted a reduction in the bacterial population in the lungs of a man with chronic colonization. The authors emphasize that the pathogen was not eliminated, but the patient showed clinical improvement, could leave the hospital, and was not at risk of death (Köhler et al. 2023). Using phages as alternative therapy raises some questions about safety and the selection of resistant bacterial strains by transduction mechanisms.

### 2.3 Phage Therapy in Osteomyelitis Caused by *P. aeruginosa*

Osteomyelitis is an inflammatory process of the bone, resulting from a fungal or bacterial infection (Pliska 2020; Simner et al. 2022). In some regions around the world, *P. aeruginosa* accounts for 10% of osteomyelitis cases and is the third most common causative agent in these bone infections (Pliska 2020). The inoculation of bacterial cultures directly into the tibia of animals appears to be the method of choice used in the study of the progression of osteomyelitis in preclinical models (Braunstein et al. 2024; Pliska 2020; Simner et al. 2022). In this review, we retrieved only clinical reports of patients who used prostheses or underwent surgery and suffered from bone infection by *P. aeruginosa*. There are promising results against MDR, XDR, and DTR strains of *P. aeruginosa* causing osteomyelitis. Intravenous administration, irrigation, or the use of compresses soaked with phages were able to control the bone infection process (Ferry et al. 2018; Racenis et al. 2022; Simner et al. 2022).

Additionally, as described for other phage therapy approaches, no study reported toxicity or adverse effects during or after the intervention with phage cocktails (Chung et al. 2023; D. Liu et al. 2021). Unfortunately, some patients died during treatment due to other complications, which made it impossible to assess the outcomes of bone infection after the removal of *P. aeruginosa* and the end of the phage intervention. The interventions occurred over 3 to 109 days, intravenously or by direct administration of phages on the lesions. All studies analyzed showed a reduction in bacterial density or total elimination of the pathogen in the treated areas and enhanced the occlusion of surgical lesions (Braunstein et al. 2024; Nadareishvili et al. 2020; Racenis et al. 2022; Simner et al. 2022).

### 2.4 Effects of Phage Therapy on Alternative Animal Models Infected With *P.* aeruginosa

Conscious scientific experimentation based on the 3 R principles (replacement, reduction, and refinement) is essential for good research practices (Serrano et al. 2023). The use of alternative *in vivo* models such as *Galleria mellonella* and *Danio rerio* larvae is encouraged due to their advantages, including ease of handling, low cost, rapid reproduction, ease of inoculation of pathogens, and responses to infection progression like those of humans (Antoine et al. 2021; Franza et al. 2024; Karthika et al. 2023; Saraceni et al. 2016; Serrano et al. 2023). The *G. mellonella* larvae have an immune system with comprising cellular and humoral immune responses (Ménard et al. 2021; Serrano et al. 2023). The cellular response is mediated by phagocytic cells called hemocytes, found within the hemolymph. The humoral response is mediated by molecules that immobilize or kill the pathogen [melanin and antimicrobial peptides (Pereira et al. 2020; Serrano et al. 2023).

Melanization of this insect is a primary characteristic of the response to environmental stress. This process begins with phagocytosis of the pathogen, stimulating the phenoloxidase pathway that leads to the polymerization of quinines to form melanin around microbes (Banfi et al. 2024; Ménard et al. 2021). The virulence of the *P. aeruginosa* turns the larvae completely dark within a few hours and, as the infection progresses, results in death (Antoine et al. 2021; Jeon and Yong 2019; Pereira et al. 2020). The administration of phage cocktails controlled the infection by *P. aeruginosa* (up to 72 h) in *G. mellonella*. However, the high virulence and pathogenicity of this bacillus affect the larvae's survival, and late administration of phages can compromise the intervention's efficacy. Incorrect injection, needle size, and lack of endotoxin quantification are characteristics that can affect the result. Therefore, new studies evaluating the best experimental standards should be conducted so that the results are due to the intervention rather than methodological variables (Kaszab et al. 2023; Kavanagh and Sheehan 2018; Marancik et al. 2020; Pereira et al. 2020).

Zebrafish (*Danio rerio*) embryos have also been used as alternative animal models due to their genomic homology with humans (> 80% of disease‐associated genes) and immunological compatibility (Cafora et al. 2019; Kaszab et al. 2023; Marancik et al. 2020; Saraceni et al. 2016; Torraca and Mostowy 2018). Furthermore, the transparency of the embryos allows for the analysis of the interaction between bacterial pathogens and phagocytic cells. In this case, genetic alterations are necessary to make the macrophages fluorescent.

Static immersion and microinjection were used to evaluate zebrafish and *P. aeruginosa* interactions. In the first case, a bacterial infection of an aquatic environment is simulated by briefly incubating zebrafish embryos in tanks containing the bacterial culture. In microinjections, the bacterial culture is inoculated directly into the Cuvier's duct, resulting in systemic infection. (Kaszab et al. 2023; Marancik et al. 2020). In both methodologies, phage cocktails reduced the bacterial population and induced survival rates above 80%. Phage cocktails were administered at doses of 10^6^ to 10^11^ PFU/mL. Intervention periods lasting between 7 h and 7 days. The phages were found to be non‐toxic and were successful in rescuing zebrafish embryos from systemic *P. aeruginosa* infection.

Zebrafish have established themselves as a robust mimetic model for understanding the progression of *P. aeruginosa* infections. However, some limitations should be discussed and considered in future studies. Initially, the virulence of the bacterial pathogen can affect survival causing premature deaths or lethargic progression with weak symptoms (Stones et al. 2017; Torraca and Mostowy 2018). Larvae in the early stages of evolution do not show a mature adaptive immune response, which can affect the course of the infection. Stones et al. (2017), suggest that the natural microbiome of *D. rerio* provides a barrier to colonization by exogenous organisms. Furthermore, in systemic infections, histological analysis will likely undergo alterations due to the greater or lesser affinity of the pathogen to the tissue analyzed. Finally, the distinct body temperature between mammals and fish is a natural limiter for bacterial growth, but this parameter is overcome by increasing the inoculum (10^9^ CFU/mL) (Franza et al. 2024; Torraca and Mostowy 2018; Whipps and Kent 2019).

## 3 Benefits of Phage Therapy for Treating *P. aeruginosa* Infections

Phage therapy offers a highly specific and adaptable strategy for managing infections caused by *P. aeruginosa*, a pathogen frequently associated with MDR, XDR, and PDR phenotypes. The reviewed studies demonstrate phage efficacy in various clinical and preclinical models, including skin wounds, pulmonary infections, osteomyelitis, keratitis, and systemic infections. In almost all models analyzed, phages significantly reduced bacterial load and, in many cases, achieved complete sterilization of infected tissues.

A major benefit of phage therapy is its host specificity, which allows the elimination of target bacteria without disrupting the commensal microbiota (Chung et al. 2023). In the models analyzed, topical, intratracheal, and intravenous routes of administration achieved bacterial control with minimal inflammatory response and no observable systemic toxicity (D. Liu et al. 2021). Moreover, phages showed compatibility with co‐administered antibiotics such as imipenem‐relebactam, enhancing therapeutic outcomes in cases where antibiotics alone had failed (Hahn et al. 2023; Köhler et al. 2023; Y. Lin et al. 2019; Olorundare et al. 2024). Phage therapy also proved to be effective in animal models that closely mimic human infection, such as BALB/c and C57BL/6 mice for pneumonia, Wistar rats for wound healing, and zebrafish and *Galleria mellonella* for systemic infection. Alternative delivery systems, including hydrogels, gauzes, and nanoformulations, have been explored to optimize phage delivery and stability in hostile environments like burn wounds and acidic gastrointestinal conditions (S. Mabrouk et al. 2022; Shafigh Kheljan et al. 2023; Yeh et al. 2020).

## 4 Limitations of Phage Therapy for Treating *P. Aeruginosa* Infections

Despite promising therapeutic results, several key barriers still limit the clinical adoption of phage therapy, including the lack of standardized treatment protocols, safety concerns related to endotoxin contamination, insufficient knowledge about host immune responses to phages, and the absence of well‐defined regulatory frameworks. First, the high specificity of bacteriophages, while advantageous for reducing off‐target effects, limits their efficacy against heterogeneous or rapidly mutating bacterial populations. Some studies required phage cocktails to broaden the host range and overcome resistance, but this introduces variability in phage selection and production protocols (Egido et al. 2022; J. Lin et al. 2022; Pires et al. 2020). Another important limitation is the lack of standardization across dosing regimens, routes of administration, and treatment durations. This reduces the possibility of comparisons between studies and can make outcomes unique depending on specific conditions, representing a challenge in defining therapeutic standards.

Moreover, while endotoxin contamination is a recognized safety concern, only a minority of studies performed quantitative analyses or reported purification. Furthermore, the immune response of the animal or human host to the phage remains poorly studied. Available evidence from the studies analyzed in this review suggests low toxicity. However, immunogenicity, neutralizing antibodies, and innate immune recognition of phages remain underexplored aspects that may influence long‐term efficacy.

There are currently no standardized regulatory guidelines specifically established for endotoxin quantification and removal in bacteriophage preparations. However, some methods can be used to quantify and remove bacterial pyrogens. Methods based on *Limulus polyfemus* amebocyte lysate can be used to quantify endotoxins in chromogenic and turbidimetric assays. Chromogenic methods measure the color intensity resulting from the cleavage of synthetic chromogen. Turbidimetric assays measure the turbidity of the medium caused by the precipitation of proteins in the blood of *L. polyfemus*, which undergo coagulation in the presence of endotoxins (Flórez et al. 2023). These components can be removed by extraction in water‐miscible solvents such as 1‐octanol, where the endotoxins are retained in the organic phase and the phages are maintained in the aqueous phase. This methodology can result in the removal of up to 99% of the endotoxins present in bacteriophage cocktails (Szermer‐Olearnik and Boratyński 2015; Van Belleghem et al. 2017).

The regulatory framework for phage therapy also remains underdeveloped in most countries, leading to uncertainty in clinical approval and production quality (Egido et al. 2022; World Health Organization 2025). Furthermore, while animal models such as mice and zebrafish provide valuable insight, they do not fully recapitulate the complexity of human immune responses or chronic infections, such as those evidenced in cystic fibrosis patients (Cafora et al. 2019; Olszak et al. 2015; Torraca and Mostowy 2018; Whipps and Kent 2019). Finally, phage resistance can emerge, particularly in monophage therapies or when bacterial populations harbor mechanisms like CRISPR‐Cas or surface modifications, necessitating continuous surveillance and updating the phage treatments (Egido et al. 2022; J. Lin et al. 2022). These limitations call for the development of robust phage libraries, harmonized protocols for phage preparation and administration, and rigorous clinical trials to evaluate efficacy and safety under standardized conditions.

## 5 Conclusions

By synthesizing heterogeneous preclinical and clinical evidence, this narrative review highlights the therapeutic promise of phage therapy against *P. aeruginosa* while emphasizing the context‐dependent nature of current approaches and the need for further translational refinement. Given the evidence that supports the efficacy of phage therapy, it is important to note the challenges that still impair its clinical integration in the field. A critical step involves the development of well‐characterized phage libraries with broad‐spectrum coverage of clinically relevant *P. aeruginosa* strains, including MDR, XDR, and PDR phenotypes. These libraries should be supported by real‐time clinical surveillance and rapid host‐range testing platforms to allow personalized or semi‐personalized phage selection within a clinically actionable timeframe. Additionally, standardization of dosing regimens and administration protocols is another urgent need. The wide variation in phage titters, treatment durations, and delivery routes noted in preclinical and clinical studies complicates reproducibility and regulatory approval. Therefore, efforts should be made to define some minimal guidelines to be developed to define several important factors, such as minimum effective phage concentrations, optimal treatment durations for different infection sites, appropriate administration routes (e.g., topical vs. systemic) and criteria for phage cocktail composition.

Pharmaceutical formulation advances are also needed to ensure stability, bioavailability, and safety of phage products. Techniques such as endotoxin removal, encapsulation in hydrogels, and aerosolized delivery systems have shown promise but require validation under Good Manufacturing Practices. Inhaled and topical formulations should be optimized for mucosal or dermal environments, while intravenous preparations must meet stringent sterility and pyrogen‐free standards to comply with regulatory expectations. In addition, preclinical model standardization is essential to the progress of phage therapy research. Finally, regulatory and policy support will be crucial for transitioning phage therapy from compassionate use to mainstream clinical practice. Establishing centralized phage banks, national production facilities, and clinical trial protocols can increase the rate of use of phage therapy in all countries.

## Author Contributions

**Gustavo Aparecido da Cunha:** conceptualization, writing – original draft, writing – review and editing, methodology, investigation, validation, formal analysis, data curation. **Raine Piva Amaral:** conceptualization, writing – original draft, writing – review and editing, methodology, data curation, investigation, validation. **Bárbara Rafaela Ferreira Aio:** writing – review and editing, data curation, validation. **Maria Fernanda Romboli Durante:** conceptualization, writing – original draft, writing – review and editing, methodology, data curation, investigation. **Luiz Cosme Cotta Malaquias:** conceptualization, writing – original draft, writing – review and editing, supervision. **Luiz Felipe Leomil Coelho:** conceptualization, validation, formal analysis, writing – review and editing, writing – original draft, funding acquisition, supervision.

## Consent

The authors have nothing to report.

## Ethics Statement

This is a narrative review and therefore ethics approval is not applicable.

## Conflicts of Interest

The authors declare no conflicts of interest.

## Data Availability

Data sharing not applicable to this article as no datasets were generated or analyzed during the current study.

## References

[mbo370303-bib-0001] Abdelaziz, M. A. , A. M. A. El‐Aziz , M. M. A. El‐Sokkary , and R. Barwa . 2024. “Characterization and Genetic Analysis of Extensively Drug‐Resistant Hospital Acquired Pseudomonas aeruginosa Isolates.” BMC Microbiology 24, no. 1: 225. 10.1186/s12866-024-03321-5. 38926687 PMC11201863

[mbo370303-bib-0002] Aghaee, B. L. , M. Khan Mirzaei , M. Y. Alikhani , A. Mojtahedi , and C. F. Maurice . 2021. “Improving the Inhibitory Effect of Phages Against Pseudomonas aeruginosa Isolated From a Burn Patient Using a Combination of Phages and Antibiotics.” Viruses 13, no. 2: 334. 10.3390/v13020334. 33670028 PMC7926668

[mbo370303-bib-0003] Almeida, G. M. F. , and L. R. Sundberg . 2020. “The Forgotten Tale of Brazilian Phage Therapy.” Lancet Infectious Diseases 20, no. 5: e90–e101. Lancet Publishing Group. 10.1016/S1473-3099(20)30060-8. 32213334

[mbo370303-bib-0004] Altamirano, F. L. G. , and J. J. Barr . 2019. “Phage Therapy in the Postantibiotic Era.” Clinical Microbiology Reviews 32, no. 2: e00066‐18. 10.1128/CMR.00066-18. 30651225 PMC6431132

[mbo370303-bib-0005] Antoine, C. , F. Laforêt , B. Blasdel , et al. 2021. “Efficacy Assessment of PEV2 Phage on Galleria Mellonella Larvae Infected With a Pseudomonas Aeruginosa Dog Otitis Isolate.” Research in Veterinary Science 136: 598–601. 10.1016/j.rvsc.2021.04.010. 33895568

[mbo370303-bib-0006] Banfi, D. , T. Bianchi , M. Mastore , and M. F. Brivio . 2024. “Optimization of Experimental Infection of the Animal Model Galleria mellonella Linnaeus 1758 (Lepidoptera: Pyralidae) With the Gram‐Positive Bacterium Micrococcus Luteus.” Insects 15, no. 8: 618. 10.3390/insects15080618. 39194822 PMC11354611

[mbo370303-bib-0007] Van Belleghem, J. D. , M. Merabishvili , B. Vergauwen , R. Lavigne , and M. Vaneechoutte . 2017. “A Comparative Study of Different Strategies for Removal of Endotoxins From Bacteriophage Preparations.” Journal of Microbiological Methods 132: 153–159. 10.1016/j.mimet.2016.11.020. 27913133

[mbo370303-bib-0008] Berlanga, M. , and R. Guerrero . 2016. “Living Together in Biofilms: The Microbial Cell Factory and Its Biotechnological Implications.” Microbial Cell Factories 15, no. 1: 165. BioMed Central Ltd. 10.1186/s12934-016-0569-5. 27716327 PMC5045575

[mbo370303-bib-0009] Braunstein, R. , G. Hubanic , O. Yerushalmy , et al. 2024. “Successful Phage‐Antibiotic Therapy of P. aeruginosa Implant‐Associated Infection in a Siamese Cat.” Veterinary Quarterly 44, no. 1: 1–9. 10.1080/01652176.2024.2350661. PMC11089911 38726795

[mbo370303-bib-0010] Cafora, M. , G. Deflorian , F. Forti , et al. 2019. “Phage Therapy Against Pseudomonas aeruginosa Infections in a Cystic Fibrosis Zebrafish Model.” Scientific Reports 9, no. 1: 1527. 10.1038/s41598-018-37636-x. 30728389 PMC6365511

[mbo370303-bib-0011] Can, K. , U. Aksu , and O. Ş. Yenen . 2018. “Investigation of Phikz Phage Therapy Against Pseudomonas aeruginosa in Mouse Pneumonia Model.” Turkish Journal of Medical Sciences 48, no. 3: 670–678. 10.3906/sag-1711-22. 29916228

[mbo370303-bib-0012] Chang, C. , X. Yu , W. Guo , et al. 2022. “Bacteriophage‐Mediated Control of Biofilm: A Promising New Dawn for the Future.” Frontiers in Microbiology 13: 825828. 10.3389/fmicb.2022.825828. 35495689 PMC9048899

[mbo370303-bib-0013] Chang, R. Y. K. , M. Y. T. Chow , Y. Wang , et al. 2022. “The Effects of Different Doses of Inhaled Bacteriophage Therapy for Pseudomonas aeruginosa Pulmonary Infections in Mice.” Clinical Microbiology and Infection 28, no. 7: 983–989. 10.1016/j.cmi.2022.01.006. 35123053

[mbo370303-bib-0014] Chegini, Z. , A. Khoshbayan , M. Taati Moghadam , I. Farahani , P. Jazireian , and A. Shariati . 2020. “Bacteriophage Therapy Against Pseudomonas aeruginosa Biofilms: A Review.” Annals of Clinical Microbiology and Antimicrobials 19, no. 1: 45. BioMed Central Ltd. 10.1186/s12941-020-00389-5. 32998720 PMC7528332

[mbo370303-bib-0015] Chen, F. , X. Cheng , J. Li , et al. 2021. “Novel Lytic Phages Protect Cells and Mice Against Pseudomonas aeruginosa Infection.” Journal of Virology 95, no. 8: e01832‐20. 10.1128/jvi.01832-20. 33472935 PMC8103703

[mbo370303-bib-0016] Chung, K. M. , S. C. Nang , and S. S. Tang . 2023. “The Safety of Bacteriophages in Treatment of Diseases Caused by Multidrug‐Resistant Bacteria.” Pharmaceuticals (Basel, Switzerland) 16, no. 10: 1347. Multidisciplinary Digital Publishing Institute (MDPI). 10.3390/ph16101347. 37895818 PMC10610463

[mbo370303-bib-0017] Cosentino, F. , P. Viale , and M. Giannella . 2023. “MDR/XDR/PDR or DTR? Which Definition Best Fits the Resistance Profile of Pseudomonas aeruginosa?” Current Opinion in Infectious Diseases 36, no. 6: 564–571. Lippincott Williams and Wilkins. 10.1097/QCO.0000000000000966. 37930070 PMC10836784

[mbo370303-bib-0018] Duplessis, C. , J. M. Warawa , M. B. Lawrenz , M. Henry , and B. Biswas . 2021. “Successful Intratracheal Treatment of Phage and Antibiotic Combination Therapy of a Multi‐Drug Resistant Pseudomonas aeruginosa Murine Model.” Antibiotics (USSR) 10, no. 8: 946. 10.3390/antibiotics10080946. PMC8388862 34438996

[mbo370303-bib-0019] Egido, J. E. , A. R. Costa , C. Aparicio‐Maldonado , P. J. Haas , and S. J. J. Brouns . 2022. “Mechanisms and Clinical Importance of Bacteriophage Resistance.” In FEMS Microbiology Reviews (46, 1). Oxford University Press. 10.1093/femsre/fuab048. PMC8829019 34558600

[mbo370303-bib-0020] Elfadadny, A. , R. F. Ragab , M. AlHarbi , et al. 2024. “Antimicrobial Resistance of Pseudomonas aeruginosa: Navigating Clinical Impacts, Current Resistance Trends, and Innovations in Breaking Therapies.” Frontiers in Microbiology 15: 1374466. 10.3389/fmicb.2024.1374466. 38646632 PMC11026690

[mbo370303-bib-0021] Ferry, T. , F. Boucher , C. Fevre , et al. 2018. “Innovations for the Treatment of a Complex Bone and Joint Infection Due to XDR Pseudomonas aeruginosa Including Local Application of a Selected Cocktail of Bacteriophages.” Journal of Antimicrobial Chemotherapy 73, no. 10: 2901–2903. Oxford University Press. 10.1093/jac/dky263. 30060002

[mbo370303-bib-0022] Ferry, T. , C. Kolenda , F. Laurent , et al. 2022. “Personalized Bacteriophage Therapy to Treat Pandrug‐Resistant Spinal Pseudomonas aeruginosa Infection.” Nature Communications 13, no. 1: 4239. 10.1038/s41467-022-31837-9. PMC9306240 35869081

[mbo370303-bib-0023] Flórez, P. , M. de Castro , D. Rodríguez , J. M. Gonzalo‐Orden , and A. Carvajal . 2023. “Intralaboratory Validation of a Kinetic Turbidimetric Assay Based on Limulus Amebocyte Lysate (LAL) for Assessing Endotoxin Activity in Cow Milk.” Animals: An Open Access Journal from MDPI 13, no. 3: 427. 10.3390/ani13030427. 36766315 PMC9913736

[mbo370303-bib-0024] Forti, F. , D. R. Roach , M. Cafora , et al. 2018. “Design of a Broad‐Range Bacteriophage Cocktail That Reduces Pseudomonas aeruginosa Biofilms and Treats Acute Infections in Two Animal Models.” Antimicrobial Agents and Chemotherapy 62, no. 6: e02573‐17. 10.1128/AAC.02573-17. 29555626 PMC5971607

[mbo370303-bib-0025] Franza, M. , R. Varricchio , G. Alloisio , et al. 2024. “Zebrafish (Danio rerio) as a Model System to Investigate the Role of the Innate Immune Response in Human Infectious Diseases.” International Journal of Molecular Sciences 25, no. 22: 12008. Multidisciplinary Digital Publishing Institute (MDPI). 10.3390/ijms252212008. 39596075 PMC11593600

[mbo370303-bib-0026] Fröhlich, E. 2024. “Animals in Respiratory Research.” International Journal of Molecular Sciences 25, no. 5: 2903. Multidisciplinary Digital Publishing Institute (MDPI). 10.3390/ijms25052903. 38474149 PMC10931704

[mbo370303-bib-0027] Fukuda, K. , W. Ishida , J. Uchiyama , et al. 2012. “Pseudomonas aeruginosa Keratitis in Mice: Effects of Topical Bacteriophage KPP12 Administration.” PLoS One 7, no. 10: e47742. 10.1371/journal.pone.0047742. 23082205 PMC3474789

[mbo370303-bib-0028] Ghanaim, A. M. , M. A. Foaad , E. Z. Gomaa , et al. 2023. “Bacteriophage Therapy as an Alternative Technique for Treatment of Multidrug‐Resistant Bacteria Causing Diabetic Foot Infection.” International Microbiology 26, no. 2: 343–359. 10.1007/s10123-022-00293-2. 36350460 PMC10148765

[mbo370303-bib-0029] Ghssein, G. , and Z. Ezzeddine . 2022. “A Review of Pseudomonas aeruginosa Metallophores: Pyoverdine, Pyochelin and Pseudopaline.” Biology 11, no. 12: 1711. MDPI. 10.3390/biology11121711. 36552220 PMC9774294

[mbo370303-bib-0030] Del Giacomo, P. , F. Raffaelli , A. R. Losito , B. Fiori , and M. Tumbarello . 2022. “XDR‐Pseudomonas aeruginosa Outside the ICU: Is There Still Place for Colistin?” Antibiotics (Basel, Switzerland) 11, no. 2: 193. 10.3390/antibiotics11020193. 35203796 PMC8868142

[mbo370303-bib-0031] Gupta, P. , H. S. Singh , V. K. Shukla , G. Nath , and S. K. Bhartiya . 2019. “Bacteriophage Therapy of Chronic Nonhealing Wound: Clinical Study.” The international journal of lower extremity wounds 18, no. 2: 171–175. 10.1177/1534734619835115. 31081402

[mbo370303-bib-0032] Hahn, A. , I. Sami , H. Chaney , et al. 2023. “Bacteriophage Therapy for Pan‐Drug‐Resistant Pseudomonas aeruginosa in Two Persons With Cystic Fibrosis.” Journal of Investigative Medicine High Impact Case Reports 11: 23247096231188243. 10.1177/23247096231188243. 37515541 PMC10387758

[mbo370303-bib-0033] Jault, P. , T. Leclerc , S. Jennes , et al. 2019. “Efficacy and Tolerability of a Cocktail of Bacteriophages to Treat Burn Wounds Infected by Pseudomonas aeruginosa (Phagoburn): A Randomised, Controlled, Double‐Blind Phase 1/2 Trial.” Lancet Infectious Diseases 19, no. 1: 35–45. 10.1016/S1473-3099(18)30482-1. 30292481

[mbo370303-bib-0034] Jeon, J. , and D. Yong . 2019. “Two Novel Bacteriophages Improve Survival in Galleria Mellonella Infection and Mouse Acute Pneumonia Models Infected With Extensively Drug‐Resistant Pseudomonas aeruginosa.” Applied and Environmental Microbiology 85, no. 9: e02900‐18. 10.1128/AEM.02900-18. 30824445 PMC6495756

[mbo370303-bib-0035] Jokar, J. , H. T. Abdulabbas , K. Javanmardi , et al. 2024. “Enhancement of Bactericidal Effects of Bacteriophage and Gentamicin Combination Regimen Against Staphylococcus aureus and Pseudomonas aeruginosa Strains in a Mice Diabetic Wound Model.” Virus Genes 60, no. 1: 80–96. 10.1007/s11262-023-02037-4. 38079060

[mbo370303-bib-0036] Jurado‐Martín, I. , M. Sainz‐Mejías , and S. McClean . 2021. “Pseudomonas aeruginosa: An Audacious Pathogen With an Adaptable Arsenal of Virulence Factors.” International Journal of Molecular Sciences 22, no. 6: 3128. MDPI AG. 10.3390/ijms22063128. 33803907 PMC8003266

[mbo370303-bib-0037] Karthika, C. , N. Malligarjunan , R. Jothi , et al. 2023. “Two Novel Phages PSPa and APPa Inhibit Planktonic, Sessile and Persister Populations of Pseudomonas aeruginosa, and Mitigate Its Virulence in Zebrafish Model.” Scientific Reports 13, no. 1: 19033. 10.1038/s41598-023-45313-x. 37923820 PMC10624879

[mbo370303-bib-0038] Kaszab, E. , D. Jiang , I. Szabó , et al. 2023. “Evaluating the In Vivo Virulence of Environmental Pseudomonas aeruginosa Using Microinjection Model of Zebrafish (Danio rerio).” Antibiotics (USSR) 12, no. 12: 1740. 10.3390/antibiotics12121740. PMC10740789 38136774

[mbo370303-bib-0039] Kaur, S. , A. Kumari , A. Kumari Negi , et al. 2021. “Nanotechnology Based Approaches in Phage Therapy: Overcoming the Pharmacological Barriers.” Frontiers in Pharmacology 12: 1–18. 10.3389/fphar.2021.699054. PMC8524003 34675801

[mbo370303-bib-0040] Kavanagh, K. , and G. Sheehan . 2018. “The Use of Galleria Mellonella Larvae to Identify Novel Antimicrobial Agents Against Fungal Species of Medical Interest.” Journal of Fungi 4, no. 3: 113. MDPI AG. 10.3390/jof4030113. 30235800 PMC6162640

[mbo370303-bib-0041] Köhler, T. , A. Luscher , L. Falconnet , et al. 2023. “Personalized Aerosolised Bacteriophage Treatment of a Chronic Lung Infection Due to Multidrug‐Resistant Pseudomonas aeruginosa.” Nature Communications 14, no. 1: 3629. 10.1038/s41467-023-39370-z. PMC10300124 37369702

[mbo370303-bib-0042] Lin, J. , F. Du , M. Long , and P. Li . 2022. “Limitations of Phage Therapy and Corresponding Optimization Strategies: A Review.” Molecules 27, no. 6: 1857. 10.3390/molecules27061857. 35335222 PMC8951143

[mbo370303-bib-0043] Lin, Y. , R. Y. K. Chang , W. J. Britton , et al. 2019. “Inhalable Combination Powder Formulations of Phage and Ciprofloxacin for P. aeruginosa Respiratory Infections.” European Journal of Pharmaceutics and Biopharmaceutics 142: 543–552. 10.1016/j.ejpb.2019.08.004. 31398437 PMC6750719

[mbo370303-bib-0044] Liu, D. , J. D. Van Belleghem , C. R. de Vries , et al. 2021. The Safety and Toxicity of Phage Therapy: A Review of Animal and Clinical Studies. Viruses (Vol. 13, 1268, 7). MDPI AG. 10.3390/v13071268. PMC8310247 34209836

[mbo370303-bib-0045] Liu, S. , H. Lu , S. Zhang , Y. Shi , and Q. Chen . 2022. “Phages Against Pathogenic Bacterial Biofilms and Biofilm‐Based Infections: A Review.” Pharmaceutics 14, no. 2: 427. 10.3390/pharmaceutics14020427. 35214158 PMC8875263

[mbo370303-bib-0074] Mabrouk, S. S. , G. R. Abdellatif , A. S. Abu Zaid , R. K. Aziz , and K. M. Aboshanab . 2022. “In Vitro and Pre‐Clinical Evaluation of Locally Isolated Phages, vB_Pae_SMP1 and vB_Pae_SMP5, Formulated as Hydrogels Against Carbapenem‐Resistant Pseudomonas aeruginosa.” Viruses 14, no. 12: 2760. 10.3390/v14122760. 36560763 PMC9780878

[mbo370303-bib-0046] Magill, S. S. , J. R. Edwards , W. Bamberg , et al. 2014. “Multistate Point‐Prevalence Survey of Health Care–Associated Infections.” New England Journal of Medicine 370, no. 13: 1198–1208. 10.1056/nejmoa1306801. 24670166 PMC4648343

[mbo370303-bib-0047] Magiorakos, A. P. , A. Srinivasan , R. B. Carey , et al. 2012. “Multidrug‐Resistant, Extensively Drug‐Resistant and Pandrug‐Resistant Bacteria: An International Expert Proposal for Interim Standard Definitions for Acquired Resistance.” Clinical Microbiology and Infection 18, no. 3: 268–281. 10.1111/j.1469-0691.2011.03570.x. 21793988

[mbo370303-bib-0048] Marancik, D. , J. Collins , J. Afema , and C. Lawrence . 2020. “Exploring the Advantages and Limitations of Sampling Methods Commonly Used in Research Facilities for Zebrafish Health Inspections.” Laboratory Animals 54, no. 4: 373–385. 10.1177/0023677219864616. 31387447

[mbo370303-bib-0049] McCallin, S. , J. C. Sacher , J. Zheng , and B. K. Chan . 2019. “Current State of Compassionate Phage Therapy.” Viruses 11, no. 4: 343. MDPI AG. 10.3390/v11040343. 31013833 PMC6521059

[mbo370303-bib-0050] Ménard, G. , A. Rouillon , V. Cattoir , and P. Y. Donnio . 2021. “Galleria mellonella as a Suitable Model of Bacterial Infection: Past, Present and Future.” Frontiers in Cellular and Infection Microbiology 11: 782733. Frontiers Media S.A. 10.3389/fcimb.2021.782733. 35004350 PMC8727906

[mbo370303-bib-0051] Mendes, J. J. , C. Leandro , S. Corte‐Real , et al. 2013. “Wound Healing Potential of Topical Bacteriophage Therapy on Diabetic Cutaneous Wounds.” Wound Repair and Regeneration 21, no. 4: 595–603. 10.1111/wrr.12056. 23755910

[mbo370303-bib-0052] Montero, M. M. , I. López Montesinos , H. Knobel , et al. 2020. “Risk Factors for Mortality Among Patients With Pseudomonas aeruginosa Bloodstream Infections: What Is the Influence of XDR Phenotype on Outcomes?” Journal of Clinical Medicine 9, no. 2: 514. 10.3390/jcm9020514. 32074947 PMC7074151

[mbo370303-bib-0053] Moussouni, M. , L. Berry , T. Sipka , M. Nguyen‐Chi , and A. B. Blanc‐Potard . 2021. “Pseudomonas Aeruginosa OprF Plays a Role in Resistance to Macrophage Clearance During Acute Infection.” Scientific Reports 11, no. 1: 359. 10.1038/s41598-020-79678-0. 33432030 PMC7801371

[mbo370303-bib-0054] Nadareishvili, L. , N. Hoyle , N. Nakaidze , et al. 2020. “Bacteriophage Therapy as a Potential Management Option for Surgical Wound Infections.” PHAGE (New Rochelle, N.Y.) 1, no. 3: 158–165. 10.1089/phage.2020.0010. 36147826 PMC9041461

[mbo370303-bib-0055] Nathwani, D. , G. Raman , K. Sulham , M. Gavaghan , and V. Menon . 2014. “Clinical and Economic Consequences of Hospital‐Acquired Resistant and Multidrug‐Resistant Pseudomonas aeruginosa Infections: A Systematic Review and Meta‐Analysis.” Antimicrobial Resistance and Infection Control 3, no. 1: 32. 10.1186/2047-2994-3-32. 25371812 PMC4219028

[mbo370303-bib-0056] Ng, Q. X. , N. Y. Ong , D. Y. X. Lee , et al. 2023. “Trends in Pseudomonas aeruginosa (P. aeruginosa) Bacteremia During the COVID‐19 Pandemic: A Systematic Review.” Antibiotics (USSR) 12, no. 2: 409. MDPI. 10.3390/antibiotics12020409. PMC9952731 36830319

[mbo370303-bib-0057] Olorundare, O. O. , N. Zrelovs , D. Kabantiyok , et al. 2024. “Isolation and Characterization of a Novel Jumbo Phage HPP‐Temi Infecting Pseudomonas aeruginosa Pa9 and Increasing Host Sensitivity to Ciprofloxacin.” Antibiotics (USSR) 13, no. 11: 1006. 10.3390/antibiotics13111006. PMC11591403 39596701

[mbo370303-bib-0058] Olszak, T. , P. Zarnowiec , W. Kaca , et al. 2015. “In Vitro and In Vivo Antibacterial Activity of Environmental Bacteriophages Against Pseudomonas Aeruginosa Strains From Cystic Fibrosis Patients.” Applied Microbiology and Biotechnology 99, no. 14: 6021–6033. 10.1007/s00253-015-6492-6. 25758956 PMC4480334

[mbo370303-bib-0059] Patel, D. R. , S. K. Bhartiya , R. Kumar , V. K. Shukla , and G. Nath . 2021. “Use of Customized Bacteriophages in the Treatment of Chronic Nonhealing Wounds: A Prospective Study.” International Journal of Lower Extremity Wounds 20, no. 1: 37–46. 10.1177/1534734619881076. 31752578

[mbo370303-bib-0060] Pereira, M. F. , C. C. Rossi , G. C. Da Silva , J. N. Rosa , and D. M. S. Bazzolli . 2020. “Galleria mellonella as an Infection Model: An In‐Depth Look at Why It Works and Practical Considerations for Successful Application.” In Pathogens and Disease (78, 8). Oxford University Press. 10.1093/femspd/ftaa056. 32960263

[mbo370303-bib-0061] Piranaghl, H. , S. Golmohammadzadeh , V. Soheili , et al. 2023. “The Potential Therapeutic Impact of a Topical Bacteriophage Preparation in Treating Pseudomonas aeruginosa‐Infected Burn Wounds in Mice.” Heliyon 9, no. 7: e18246. 10.1016/j.heliyon.2023.e18246. 37539104 PMC10393627

[mbo370303-bib-0062] Pires, D. P. , A. R. Costa , G. Pinto , L. Meneses , and J. Azeredo . 2020. “Current Challenges and Future Opportunities of Phage Therapy.” FEMS Microbiology Reviews 44, no. 6: 684–700. 10.1093/femsre/fuaa017. 32472938

[mbo370303-bib-0063] Pirnay, J. P. , S. Djebara , G. Steurs , et al. 2024. “Personalized Bacteriophage Therapy Outcomes for 100 Consecutive Cases: A Multicentre, Multinational, Retrospective Observational Study.” Nature Microbiology 9, no. 6: 1434–1453. 10.1038/s41564-024-01705-x. PMC11153159 38834776

[mbo370303-bib-0064] Pliska, N. N. 2020. “Pseudomonas Aeruginosa as the Main Causative Agent of Osteomyelitis and Its Susceptibility to Antibiotics.” Drug Research 70, no. 6: 280–285. 10.1055/a-1150-2372. 32303092

[mbo370303-bib-0065] Racenis, K. , D. Rezevska , M. Madelane , et al. 2022. “Use of Phage Cocktail BFC 1.10 in Combination With Ceftazidime‐Avibactam in the Treatment of Multidrug‐Resistant Pseudomonas aeruginosa Femur Osteomyelitis—A Case Report.” Frontiers in Medicine 9: 851310. 10.3389/fmed.2022.851310. 35547216 PMC9081798

[mbo370303-bib-0066] Saraceni, P. R. , A. Romero , A. Figueras , and B. Novoa . 2016. “Establishment of Infection Models in Zebrafish Larvae (Danio rerio) to Study the Pathogenesis of Aeromonas hydrophila.” Frontiers in Microbiology 7: 1219. 10.3389/fmicb.2016.01219. 27540375 PMC4972827

[mbo370303-bib-0067] Sathe, N. , P. Beech , L. Croft , C. Suphioglu , A. Kapat , and E. Athan . 2023. “Pseudomonas aeruginosa: Infections and Novel Approaches to Treatment ‘Knowing the Enemy’ the Threat of Pseudomonas Aeruginosa and Exploring Novel Approaches to Treatment.” Infectious Medicine 2, no. 3: 178–194. Elsevier B.V. 10.1016/j.imj.2023.05.003. 38073886 PMC10699684

[mbo370303-bib-0068] Schalk, I. J. , and L. Guillon . 2013. “Pyoverdine Biosynthesis and Secretion in Pseudomonas aeruginosa: Implications for Metal Homeostasis.” Environmental Microbiology 15, no. 6: 1661–1673. 10.1111/1462-2920.12013. 23126435

[mbo370303-bib-0069] Schalk, I. J. , C. Hennard , C. Dugave , K. Poole , M. A. Abdallah , and F. Pattus . 2001. “Iron‐Free Pyoverdin Binds to Its Outer Membrane Receptor FpvA in Pseudomonas aAeruginosa: A New Mechanism for Membrane Iron Transport.” Molecular Microbiology 39, no. 2: 351–361. 10.1046/j.1365-2958.2001.02207.x. 11136456

[mbo370303-bib-0070] Serrano, I. , C. Verdial , L. Tavares , and M. Oliveira . 2023. “The Virtuous Galleria mellonella Model for Scientific Experimentation.” Antibiotics (USSR) 12, no. 3: 505. MDPI. 10.3390/antibiotics12030505. PMC10044286 36978373

[mbo370303-bib-0071] Shafigh Kheljan, F. , F. Sheikhzadeh Hesari , M. Aminifazl , M. Skurnik , S. Goladze , and G. Zarrini . 2023. “Design of Phage‐Cocktail–Containing Hydrogel for the Treatment of Pseudomonas aeruginosa–Infected Wounds.” Viruses 15, no. 3: 803. 10.3390/v15030803. 36992511 PMC10051971

[mbo370303-bib-0072] Shi, Q. , C. Huang , T. Xiao , Z. Wu , and Y. Xiao . 2019. “A Retrospective Analysis of Pseudomonas Aeruginosa Bloodstream Infections: Prevalence, Risk Factors, and Outcome in Carbapenem‐Susceptible and ‐Non‐Susceptible Infections.” Antimicrobial Resistance & Infection Control 8, no. 1: 68. 10.1186/s13756-019-0520-8. 31057792 PMC6485151

[mbo370303-bib-0073] Simner, P. J. , J. Cherian , G. A. Suh , et al. 2022. “Combination of Phage Therapy and Cefiderocol to Successfully Treat Pseudomonas aeruginosa Cranial Osteomyelitis.” JAC‐Antimicrobial Resistance 4, no. 3: dlac046. 10.1093/jacamr/dlac046. 35529052 PMC9071546

[mbo370303-bib-0075] Stones, D. H. , A. G. J. Fehr , L. Thompson , et al. 2017. “Zebrafish (Danio rerio) as a Vertebrate Model Host To Study Colonization, Pathogenesis, and Transmission of Foodborne Escherichia Coli O157.” mSphere 2, no. 5: e00365‐17. 10.1128/mspheredirect.00365-17. 28959735 PMC5607324

[mbo370303-bib-0076] Strathdee, S. A. , G. F. Hatfull , V. K. Mutalik , and R. T. Schooley . 2023. “Phage Therapy: From Biological Mechanisms to Future Directions.” Cell 186, no. 1: 17–31. Elsevier B.V. 10.1016/j.cell.2022.11.017. 36608652 PMC9827498

[mbo370303-bib-0077] Sullivan, T. P. , W. H. Eaglstein , S. C. Davis , and P. Mertz . 2001. “The Pig as a Model for Human Wound Healing.” Wound Repair and Regeneration 9, no. 2: 66–76. 10.1046/j.1524-475x.2001.00066.x. 11350644

[mbo370303-bib-0078] Summers, W. C. 2012. “The Strange History of Phage Therapy.” Bacteriophage 2, no. 2: 130–133. 10.4161/bact.20757. 23050223 PMC3442826

[mbo370303-bib-0079] Szermer‐Olearnik, B. , and J. Boratyński . 2015. “Removal of Endotoxins From Bacteriophage Preparations by Extraction With Organic Solvents.” PLoS One 10, no. 3: e0122672. 10.1371/journal.pone.0122672. 25811193 PMC4374689

[mbo370303-bib-0080] Teney, C. , J. C. Poupelin , T. Briot , et al. 2024. “Phage Therapy in a Burn Patient Colonized With Extensively Drug‐Resistant Pseudomonas aeruginosa Responsible for Relapsing Ventilator‐Associated Pneumonia and Bacteriemia.” Viruses 16, no. 7: 1080. 10.3390/v16071080. 39066242 PMC11281479

[mbo370303-bib-0081] Torraca, V. , and S. Mostowy . 2018. “Zebrafish Infection: From Pathogenesis to Cell Biology.” Trends in Cell Biology 28, no. 2: 143–156. Elsevier Ltd. 10.1016/j.tcb.2017.10.002. 29173800 PMC5777827

[mbo370303-bib-0082] Waters, E. M. , D. R. Neill , B. Kaman , et al. 2017. “Phage Therapy Is Highly Effective Against Chronic Lung Infections With Pseudomonas aeruginosa.” Thorax 72, no. 7: 666–667. 10.1136/thoraxjnl-2016-209265. 28265031 PMC5520275

[mbo370303-bib-0083] Weiner, L. M. , A. K. Webb , B. Limbago , et al. 2016. “Antimicrobial‐Resistant Pathogens Associated With Healthcare‐Associated Infections: Summary of Data Reported to the National Healthcare Safety Network at the Centers for Disease Control and Prevention, 2011‐2014.” Infection Control & Hospital Epidemiology 37, no. 11: 1288–1301. 10.1017/ice.2016.174. 27573805 PMC6857725

[mbo370303-bib-0084] Whipps, C. M. , and M. L. Kent . 2019. “Bacterial and Fungal Diseases of Zebrafish.” In The Zebrafish in Biomedical Research: Biology, Husbandry, Diseases, and Research Applications, 495–508. Elsevier. 10.1016/B978-0-12-812431-4.00041-5.

[mbo370303-bib-0085] World Health Organization . 2024. Bacterial Pathogens of Public Health Importance to Guide Research, Development and Strategies to Prevent and Control Antimicrobial Resistance. https://www.who.int/publications/i/item/9789240093461.

[mbo370303-bib-0086] World Health Organization . 2025, February 17. Bacteriophages and Their Use in Combating Antimicrobial Resistance.

[mbo370303-bib-0087] World Health Organization and the European Centre for Disease Prevention Control . 2022. Antimicrobial Resistance Surveillance in Europe. 10.2900/112339.

[mbo370303-bib-0088] Yaeger, L. N. , M. R. M. Ranieri , J. Chee , et al. 2024. “A Genetic Screen Identifies a Role for oprF in Pseudomonas aeruginosa Biofilm Stimulation by Subinhibitory Antibiotics.” NPJ Biofilms and Microbiomes 10, no. 1: 30. 10.1038/s41522-024-00496-7. 38521769 PMC10960818

[mbo370303-bib-0089] Yang, X. , A. Haque , S. Matsuzaki , T. Matsumoto , and S. Nakamura . 2021. “The Efficacy of Phage Therapy in a Murine Model of Pseudomonas aeruginosa Pneumonia and Sepsis.” Frontiers in Microbiology 12: 682255. 10.3389/fmicb.2021.682255. 34290683 PMC8287650

[mbo370303-bib-0090] Yeh, Y. C. , T. H. Huang , S. C. Yang , C. C. Chen , and J. Y. Fang . 2020. “Nano‐Based Drug Delivery or Targeting to Eradicate Bacteria for Infection Mitigation: A Review of Recent Advances.” Frontiers in Chemistry 8: 286. 10.3389/fchem.2020.00286. 32391321 PMC7193053

[mbo370303-bib-0091] Zhang, Y. , B. Meng , X. Wei , et al. 2021. “Evaluation of Phage Therapy for Pulmonary Infection of Mouse by Liquid Aerosol‐Exposure Pseudomonas aeruginosa.” Infection and Drug Resistance 14: 4457–4469. 10.2147/IDR.S326230. 34737586 PMC8558430

